# Oxidized-Lipid Signaling and Ferroptosis as Downstream Mechanisms of Titanium-Associated Peri-Implant Bone Loss

**DOI:** 10.3390/antiox15091174

**Published:** 2026-09-16

**Authors:** Łukasz Woźniak, Bożena Antonowicz, Żaneta Anna Mierzejewska, Ewelina Kosicka, Jérôme R. Lechien, Luigi Angelo Vaira, Jan Borys

**Affiliations:** 1 Department of Dental Surgery, Medical University of Bialystok, M. Sklodowskiej-Curie 24A, 15-276 Bialystok, Poland; bozena.antonowicz@umb.edu.pl; 2 Institute of Biomedical Engineering, Faculty of Mechanical Engineering, Bialystok University of Technology, Wiejska 45C, 15-351 Bialystok, Poland; 3 Department of Information Technology and Robotics in Production, Faculty of Mechanical Engineering, Lublin University of Technology, Nadbystrzycka 36, 20-618 Lublin, Poland; e.kosicka@pollub.pl; 4 Department of Surgery, UMONS Research Institute for Health Sciences and Technology, University of Mons, Place du Parc 20, 7000 Mons, Belgium; jerome.lechien@umons.ac.be; 5 Department of Otolaryngology-Head & Neck Surgery, Foch Hospital, Paris Saclay University, 92150 Suresnes, France; 6 Maxillofacial Surgery Operative Unit, Department of Medicine, Surgery and Pharmacy, University of Sassari, 07100 Sassari, Italy; lavaira@uniss.it; 7 Department of Maxillofacial and Plastic Surgery, Medical University of Bialystok, M. Sklodowskiej-Curie 24A, 15-276 Bialystok, Poland; jan.borys@pb.edu.pl

**Keywords:** titanium dental implants, lipid peroxidation, ferroptosis, GPX4, oxidized phospholipids, RANKL/OPG, peri-implantitis, osteoimmunology, specialized pro-resolving mediators

## Abstract

Titanium and its alloys remain the dominant materials in oral implantology, but degradation products released through corrosion, tribocorrosion, and wear are biologically active. This critical narrative review examines oxidized-lipid signaling and ferroptosis as candidate downstream mechanisms connecting titanium-associated redox dysregulation with peri-implant bone loss. A targeted literature search of PubMed, Web of Science, and Scopus was updated through 2 August 2026 and integrated evidence from dental peri-implant studies, titanium-particle osteolysis models, and broader skeletal research. Titanium-derived particles can promote mitochondrial and non-mitochondrial reactive oxygen species, membrane phospholipid peroxidation, reactive aldehyde formation, and oxidized-phospholipid signaling. Experimental titanium-particle models demonstrate GPX4 repression and osteoblast ferroptosis, while skeletal studies indicate that ferroptotic dysfunction of osteoblasts and osteocytes can impair mineralization, increase the RANKL/OPG ratio, and favor osteoclastogenesis. Human peri-implant fluid data provide emerging but non-diagnostic evidence, including altered GPX4 and malondialdehyde levels. Specialized pro-resolving mediators may counterbalance the destructive lipid-peroxidation branch. Current evidence supports a biologically plausible and experimentally testable pathway rather than established causation in human dental peri-implant tissues. Direct tissue-level confirmation using redox lipidomics, iron mapping, pathway-specific rescue experiments, and spatial osteoimmune profiling is required.

## 1. Introduction

Titanium and titanium-based alloys have long been regarded as biologically inert biomaterials capable of maintaining long-term mechanical and chemical stability within physiological environments. This assumption is increasingly challenged: titanium undergoes progressive surface degradation through electrochemical corrosion, tribocorrosion, and biofilm-mediated processes, releasing ions and micro- and nanoscale particles into peri-implant and systemic compartments [1,2,3,4]. Once released, these degradation products are biologically active, capable of modulating mitochondrial function, redox homeostasis, inflammasome activity, cytokine networks, and bone-remodeling pathways [5,6].

An additional concept relevant to titanium degradation is metallosis, broadly understood as the local accumulation of metal-derived particles and ions within peri-implant tissues following mechanical wear, corrosion, tribocorrosion, or biofilm-associated disruption of the passive oxide layer. Titanium-containing particles have been identified in peri-implant soft tissues, macrophages, connective tissue, and bone, with higher particle burdens reported at some peri-implantitis sites. Such deposition may be accompanied by foreign-body and inflammatory responses and, in clinically apparent cases, by dark discoloration of the surrounding tissues. However, the presence of titanium debris should not itself be equated with disease causation, because titanium particles can also be detected around clinically healthy implants and current evidence does not establish a simple unidirectional relationship between metallosis and peri-implant bone loss [2,4,7,8,9,10,11,12].

Material selection in implant dentistry is also influenced by practical and economic considerations. Ceramic (particularly zirconia-based) implants provide a metal-free alternative to titanium, but titanium remains the reference material supported by a substantially longer clinical history, extensive manufacturing standardization, and broad availability across implant systems. Treatment costs may therefore influence material selection in individual healthcare settings; however, robust comparative cost-effectiveness data for titanium versus zirconia implant therapy remain limited. Economic considerations should consequently be regarded as context-dependent factors in clinical adoption rather than as evidence for or against the biological safety of either material.

In a previous critical review, we proposed that oxidative stress—rather than mechanical overload or classical metal hypersensitivity alone—functions as a central and unifying driver of adverse tissue reactions to titanium-based dental implants, with pathology arising not merely from increased reactive oxygen species (ROS) production but from failure of local antioxidant defenses [1]. That work established the upstream logic of the problem: why the peri-implant interface becomes a redox-dysregulated microenvironment. It did not, however, resolve the downstream question that determines clinical outcome—namely, through which molecular effector mechanisms a redox signal is converted into progressive bone destruction.

The present review is deliberately scoped to that downstream question. Rather than re-establishing ROS as a driver, we take it as the starting point and examine oxidized-lipid signaling as a candidate effector axis linking titanium-associated oxidative stress to peri-implant osteoimmune dysregulation. Membrane lipids are among the most reactive targets of ROS, and their peroxidation generates a cascade of bioactive products—malondialdehyde (MDA), 4-hydroxynonenal (4-HNE), oxidized phospholipids, and isoprostanes—that are not passive markers of injury but active signaling molecules capable of modifying proteins, amplifying NF-κB-dependent inflammation, and reprogramming bone-cell behavior [13,14].

Within this lipid-centered framework, we give particular attention to ferroptosis, an iron-dependent form of regulated cell death driven specifically by the accumulation of lipid peroxides and governed by the system Xc^−^–glutathione–glutathione peroxidase 4 (GPX4) axis [15,16]. Ferroptosis is mechanistically distinct from apoptosis, necroptosis, and pyroptosis, and is emerging as an important contributor to skeletal pathology: inactivation of GPX4, iron overload, and Fenton-driven radical generation trigger lipid-peroxidation cascades that impair osteoblast survival and differentiation and shift the balance toward bone resorption [17,18]. We argue that the peri-implant microenvironment—characterized by particle-driven ROS, catalytically active metal surfaces, local iron availability, and chronic inflammation—constitutes a credible mechanistic bridge between titanium degradation and dysregulation of the RANKL/OPG axis and osteoclastogenesis. A direct metallosis–ferroptosis relationship has not yet been demonstrated in human dental peri-implant tissues. Nevertheless, the two processes can be mechanistically connected. Metallosis represents an upstream material-degradation state characterized by the accumulation of metal-derived particles and ions, persistent inflammatory signaling, and redox disturbance, whereas ferroptosis represents a downstream cellular response defined by iron-dependent phospholipid peroxidation and failure of anti-ferroptotic defenses. Importantly, titanium particles have already been shown experimentally to repress GPX4 and induce osteoblast ferroptosis and peri-implant osteolysis [19]. Thus, metallosis should not be considered synonymous with ferroptosis, but it may create a redox and inflammatory microenvironment permissive for ferroptotic injury. Whether this sequence occurs in human dental peri-implant tissues remains to be established.

Crucially, the biology of oxidized lipids is not uniformly destructive. The same oxidative remodeling that yields pro-inflammatory species also generates specialized pro-resolving mediators (SPMs) and other oxylipins involved in inflammatory resolution and tissue homeostasis [20]. This duality reframes oxidized lipids as a potential therapeutic lever rather than merely a form of collateral damage, and motivates redox- and lipid-targeted strategies—GPX4 preservation, iron modulation, ferroptosis inhibition, and pro-resolving lipid signaling—as candidate approaches for protecting peri-implant bone.

**Figure 1:**
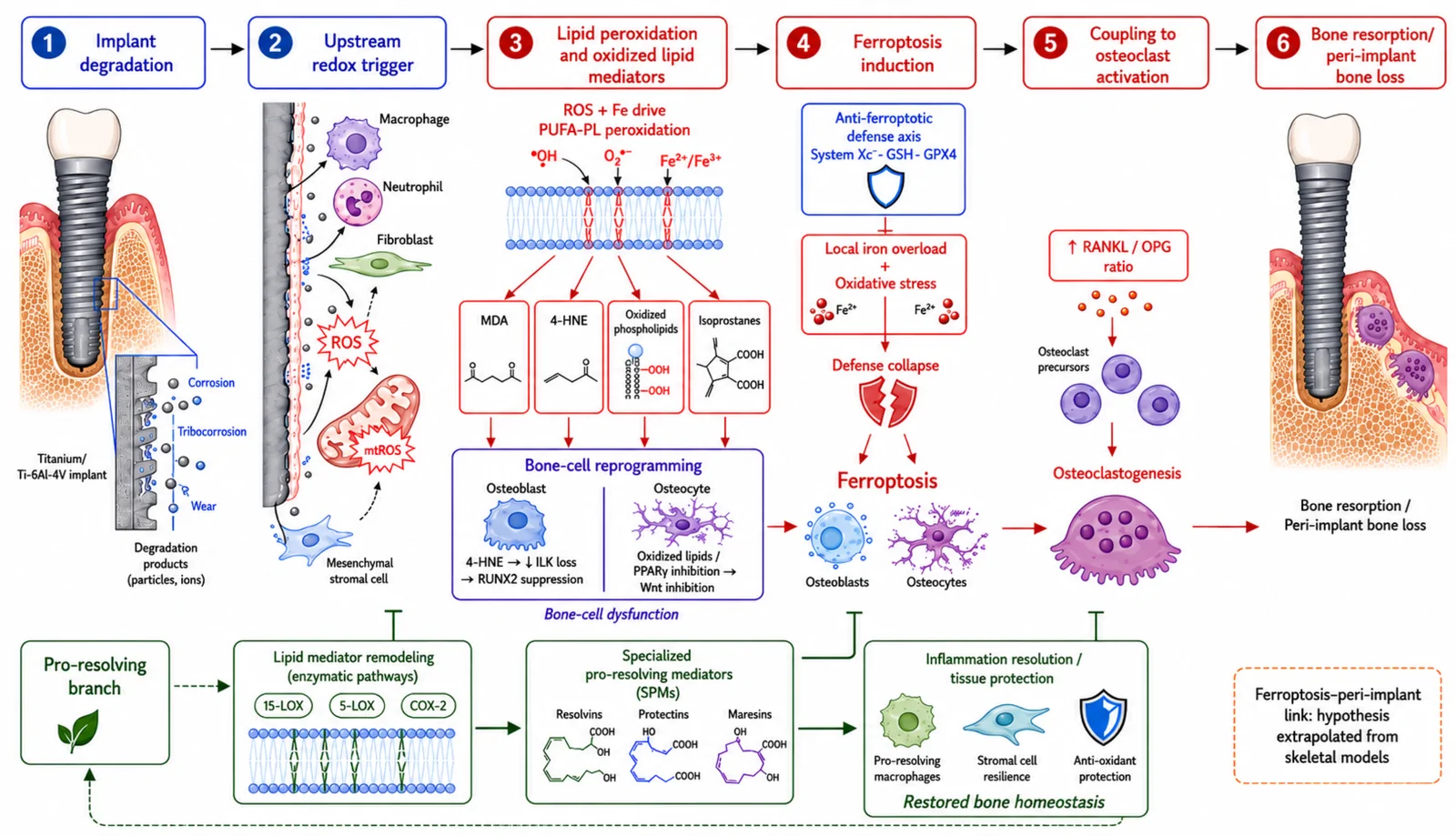
Proposed oxidized-lipid–ferroptosis effector axis linking titanium degradation to peri-implant bone loss. Titanium and Ti-6Al-4V degradation products can promote reactive oxygen species (ROS) generation and mitochondrial redox dysfunction at the implant–tissue interface. ROS and locally available iron promote peroxidation of membrane polyunsaturated fatty acids, generating reactive aldehydes, oxidized phospholipids, and ferroptotic lipid peroxides. These products can impair osteogenic signaling through the 4-HNE–ILK–RUNX2 and oxidized-lipid–PPARγ–Wnt pathways and, when system Xc^−^–GSH–GPX4 defenses become insufficient, may promote ferroptotic injury of osteoblasts and osteocytes. Ferroptotic dysfunction of bone cells may subsequently favor increased RANKL/OPG signaling, osteoclastogenesis, and bone resorption. A parallel pro-resolving lipid-mediator branch may counterbalance these destructive processes. Black arrows indicate the overall sequential progression of the proposed mechanistic axis. Red arrows indicate pro-oxidative, pro-ferroptotic, and bone-destructive signaling pathways, whereas green arrows and connecting lines represent counter-regulatory pro-resolving pathways promoting restoration of tissue homeostasis. Green blunt-ended lines indicate inhibitory or counteracting effects. Dashed green arrows indicate proposed indirect or feedback relationships within the pro-resolving branch. Solid arrows indicate relationships supported by experimental evidence, whereas integration of these individual mechanisms into a complete titanium–ferroptosis–peri-implant bone-loss pathway in human peri-implant tissues remains a mechanistic hypothesis requiring direct validation. Original schematic developed by the authors based on published evidence [1,13,14,15,16,17,18,19,20,29,36,40,64,65,69,70,71]. Created with BioRender.com.

The purpose of this review is therefore to (i) synthesize current evidence on lipid peroxidation as an effector mechanism in titanium-associated peri-implant pathology; (ii) develop the case for ferroptosis as an underappreciated, iron- and lipid-dependent mode of bone-cell death at the implant interface; (iii) examine the counterbalancing role of pro-resolving lipid mediators; and (iv) critically appraise the methodological and evidentiary limitations that constrain causal interpretation. Where the upstream redox logic is required as background, we refer the reader to our earlier analysis [1] rather than reproducing it here. An overview of the proposed effector axis is provided in Figure 1 (Section 9). Crucially, our aim is not to present ferroptosis as an established mechanism of human peri-implant bone loss, but to evaluate whether the available evidence justifies its consideration as a testable downstream effector pathway and to identify the experimental steps required for its direct validation.

## 2. Materials and Methods

This work is a critical narrative review supported by a targeted, structured literature search. PubMed, Web of Science, and Scopus were searched without a lower date limit, and the search was updated on 2 August 2026. Search concepts covered titanium and Ti-6Al-4V degradation; dental and orthopedic implant osteolysis; oxidative stress and lipid peroxidation; ferroptosis and its regulatory systems; iron metabolism; bone-cell biology; RANKL/OPG signaling; osteoclastogenesis; and specialized pro-resolving mediators. Representative Boolean combinations included: (titanium OR titanium dioxide OR Ti-6Al-4V) AND (implant OR peri-implantitis OR osteolysis) AND (oxidative stress OR lipid peroxidation OR ferroptosis OR GPX4 OR SLC7A11 OR iron) AND (osteoblast OR osteocyte OR osteoclast OR RANKL OR bone). Reference lists of key primary studies and reviews were screened to identify additional mechanistically relevant publications.

Experimental, clinical, and translational studies were considered when they addressed titanium degradation, oxidized-lipid biology, ferroptosis, iron-dependent redox mechanisms, or bone-cell responses relevant to implant-associated osteolysis. Priority was given to primary mechanistic studies, human peri-implant investigations, and authoritative reviews of ferroptosis and lipid oxidation. Purely mechanical or metallurgical studies without a biological endpoint were excluded. Because this review was designed as a mechanistic synthesis rather than a systematic review, no protocol was registered, no PRISMA flow diagram was prepared, and no formal risk-of-bias assessment or meta-analysis was performed. Evidence was interpreted hierarchically as (i) human dental peri-implant observations; (ii) titanium-particle or periprosthetic osteolysis models; and (iii) extrapolation from broader skeletal and cell-biological studies. This hierarchy is stated explicitly wherever causal inference remains limited.

## 3. Titanium Degradation and the Redox Origin of Lipid Injury

### 3.1. The Redox-Active Peri-Implant Interface

Titanium and titanium-alloy degradation products—released through corrosion, tribocorrosion, and wear—are not biologically inert; internalized by phagocytes and stromal cells, they promote ROS generation through phagocyte respiratory burst, NADPH-oxidase activity, lysosomal destabilization, and surface-catalyzed redox reactions, while overwhelming glutathione-, superoxide-dismutase-, catalase-, and Nrf2-dependent defenses [1,5,7,8,9,10]. We have previously analyzed these upstream ROS-generating and antioxidant-failure mechanisms in detail [1]; rather than re-deriving them, we take the resulting redox imbalance as the starting condition and follow its proximate biochemical consequence—oxidative modification of membrane lipids. The single mechanistic point that must be carried forward is that this redox burden is chronic and self-amplifying, because oxidative stress accelerates further material degradation, sustaining a continuous supply of oxidizing species at the interface [1].

### 3.2. Mitochondria as an Important Source of Peroxidizing Radicals

Among the intracellular sources of ROS relevant to lipid injury, mitochondria are an important—but not universally obligatory—contributor. Following cellular uptake, titanium and TiO_2_-derived particles can perturb mitochondrial membrane potential, oxidative phosphorylation, organelle dynamics, and electron transport, thereby increasing mitochondrial ROS (mtROS) production [21,22,23,24,25]. In fibroblasts exposed to differently treated Ti-6Al-4V surfaces, mitochondrial redox balance varied with surface condition [23]. Mitochondrial membranes contain cardiolipin and other oxidation-sensitive phospholipids, making them both a source and a target of lipid-peroxidation reactions [26]. However, ferroptotic lipid damage can also be initiated at the plasma membrane, endoplasmic reticulum, and other membrane compartments. Accordingly, mitochondrial dysfunction is best interpreted as a context-dependent amplifier and spatial organizer of the oxidized-lipid response rather than as an obligatory first committed step.

### 3.3. Lipid Peroxidation: From Reactive Oxygen Species to Reactive Lipids

ROS generated at the peri-implant interface do not act primarily on isolated targets; their most consequential reactions occur at the polyunsaturated fatty acids (PUFAs) of cellular and organellar membranes. Peroxidation of PUFAs proceeds through hydrogen abstraction and oxygen insertion, generating lipid hydroperoxides that decompose into reactive electrophilic aldehydes, principally MDA and 4-HNE, alongside oxidized phospholipids and isoprostanes [13,27,28,29,30,31,32,33,34]. This process can proceed non-enzymatically, driven by iron-catalyzed Fenton chemistry, or enzymatically through lipoxygenases such as ALOX15; both routes have been documented in skeletal tissue and linked to impaired bone formation [32]. Unlike the transient radicals that initiate them, these products are comparatively stable and diffusible, allowing the oxidative signal to propagate beyond its site of origin.

### 3.4. Oxidized Lipids as Signaling Molecules That Reprogram Bone Cells

MDA and 4-HNE are frequently treated as inert biomarkers of oxidative injury, yet both are chemically reactive and biologically active. 4-HNE forms Michael adducts with cysteine, histidine, and lysine residues, modifying the function of enzymes, receptors, and transcription factors, and at sustained concentrations amplifies NF-κB-dependent inflammatory transcription [14,35]. Beyond generic inflammation, these species act directly on bone-cell fate. In osteoblasts, 4-HNE—an end-product of phospholipid peroxidation—binds integrin-linked kinase and triggers its degradation, disrupting RUNX2 signaling and inhibiting osteoblastogenesis; the same study showed that phospholipid peroxidation downstream of reduced GPX4 impairs osteoblast function [34]. In parallel, oxidized lipids acting as PPARγ ligands divert mesenchymal progenitors toward adipogenesis and attenuate pro-osteogenic Wnt/β-catenin signaling, an effect reproduced by forced lipoxygenase expression [36]. Oxidized phospholipids further act as ligands for pattern-recognition and scavenger receptors, modulating innate immune activation [37]. Lipid peroxidation is therefore not the end of the oxidative cascade but a signal-transduction step that translates a diffuse redox imbalance into defined molecular species with specific bone-cell targets.

Consistent with this, elevated MDA has been documented in saliva and peri-implant crevicular fluid in peri-implant disease [35], and circulating markers of lipid and protein oxidation are altered in patients carrying titanium implants [38]. The principal oxidized-lipid species, their origins, and their proposed bone-related actions are compared in Table 1.

### 3.5. Membrane Remodeling Determines Susceptibility to Ferroptotic Lipid Damage

The susceptibility of a cell to ferroptosis is determined not only by the magnitude of ROS generation but also by the molecular composition of its membranes. Long-chain polyunsaturated fatty acids are activated by acyl-CoA synthetase long-chain family member 4 (ACSL4) and incorporated into phospholipids by remodeling enzymes including LPCAT3. Arachidonoyl- and adrenoyl-phosphatidylethanolamines are particularly important substrates for ferroptotic oxidation [29,30,31,32]. Lipoxygenase-dependent oxidation, including reactions facilitated by 15-LOX–PEBP1 complexes, can generate stereospecific hydroperoxy-phospholipids, whereas non-enzymatic radical propagation produces a broader oxidized-lipid spectrum [30,32]. This distinction is experimentally relevant: total MDA or bulk ROS measurements cannot identify the specific phospholipid species that execute ferroptosis. Redox lipidomics capable of resolving oxidized phosphatidylethanolamine species is therefore needed to distinguish generalized oxidative injury from a bona fide ferroptotic program at the implant–bone interface.

### 3.6. The Dual Biology of Oxidized Lipids

Critically, oxidative remodeling of membrane lipids is not uniformly destructive. The same enzymatic and non-enzymatic machinery that yields pro-inflammatory aldehydes also generates oxygenated derivatives—lipoxins, resolvins, protectins, and related oxylipins collectively termed SPMs—that actively terminate inflammation and promote tissue repair [20,43,44,45,46]. Whether a given peri-implant microenvironment tips toward injury or resolution therefore depends not only on the quantity of ROS but on the qualitative balance of the lipid products formed. This duality is central to the present review: it reframes oxidized lipids as a potential therapeutic lever rather than as collateral damage, and it distinguishes the destructive branch—culminating in ferroptosis—from the resolving branch that may protect peri-implant bone.

## 4. Ferroptosis: An Iron- and Lipid-Dependent Mode of Bone-Cell Death at the Peri-Implant Interface

### 4.1. Definition and Mechanistic Distinctiveness

Ferroptosis is an iron-dependent, non-apoptotic form of regulated cell death driven by the accumulation of lethal lipid peroxides, first defined by Dixon and colleagues [15,47,48,49,50]. It is mechanistically and morphologically distinct from apoptosis, necroptosis, and pyroptosis: it is not blocked by caspase or necroptosis inhibitors but is specifically suppressed by iron chelators and lipophilic radical-trapping antioxidants such as ferrostatin-1 [15,18]. This distinction is clinically relevant, because titanium particles recovered from patients with maxillofacial bone fixations have been associated with elevated TNF-α and caspase-3, markers that capture inflammatory and apoptotic responses but would not detect a concurrent ferroptotic component [51]. Morphologically it is characterized by mitochondrial shrinkage with increased membrane density and loss of cristae, in the presence of an intact nucleus [16,52]. These distinguishing features, which matter for interpreting peri-implant cell-death data, are summarized in Table 2. Its execution is governed by the balance between PUFA peroxidation and the cell’s capacity to neutralize lipid hydroperoxides.

### 4.2. The System Xc^−^–GSH–GPX4 Axis and Its Collapse Under Oxidative Load

The central defense against ferroptosis is the system Xc^−^–glutathione–GPX4 axis. The cystine/glutamate antiporter system Xc^−^ (SLC7A11) imports cystine for glutathione (GSH) synthesis; GSH is the cofactor that enables GPX4 to reduce phospholipid hydroperoxides to non-toxic alcohols [16,17,55,56]. When GSH is depleted or GPX4 inactivated, lipid hydroperoxides accumulate unchecked and propagate membrane peroxidation to a lethal threshold. This axis links ferroptosis directly to the antioxidant failure we described previously as the pivot of titanium redox pathology [1]: the peri-implant microenvironment is characterized precisely by chronic GSH consumption and dysregulated Nrf2-dependent antioxidant transcription, the conditions under which GPX4 protection is most likely to fail. In osteoblasts specifically, the Nrf2/HO-1 pathway sustains GPX4 and system Xc^−^, and its failure precipitates ferroptotic loss of osteogenic capacity [54,55]. Parallel GSH-independent defenses—the FSP1–CoQ10–NADPH and DHODH–CoQ10 systems—provide backup that may be similarly overwhelmed [16,57,58,59,60,61].

### 4.3. Iron Availability at the Peri-Implant Interface

Ferroptosis depends on the availability of redox-active iron rather than on total tissue iron content. In the peri-implant microenvironment, microhemorrhage during implant placement, recurrent mucosal bleeding, and erythrocyte extravasation may provide a biologically relevant source of heme-bound iron. Following erythrophagocytosis by macrophages, hemoglobin is degraded within the phagolysosomal compartment and heme is subsequently catabolized by heme oxygenase-1 (HO-1), releasing ferrous iron (Fe^2+^). This iron can enter the cytosolic labile iron pool, be sequestered in ferritin, or be exported through ferroportin. Experimental evidence from hemorrhage-associated inflammatory models demonstrates that erythrophagocytosis can increase the macrophage labile iron pool, promote lipid peroxidation, and induce ferroptotic cell death [62,63].

Inflammation may further shift this balance toward intracellular iron retention. Hepcidin promotes the internalization and degradation of ferroportin, thereby limiting cellular iron export, particularly from macrophages. At the same time, ferritin provides a major protective iron-storage compartment; however, NCOA4-mediated ferritinophagy can remobilize ferritin-bound iron and increase the redox-active intracellular pool. Transferrin-bound Fe^3+^ represents another source: following TFR1-mediated endocytosis, Fe^3+^ is reduced to Fe^2+^ in endosomes and transferred to the cytosol. This pathway is particularly relevant to bone remodeling because TFR1-mediated iron uptake increases in osteoclast-lineage cells and supports mitochondrial metabolism, cytoskeletal organization, and bone-resorptive activity [64,65,66]. Collectively, hemorrhage, erythrocyte turnover, altered hepcidin–ferroportin signaling, ferritin turnover, and increased transferrin-receptor-mediated uptake can increase the local labile Fe^2+^ pool without necessarily producing a proportional increase in total tissue iron. This distinction is important because Fe^2+^, rather than total iron, drives Fenton-type radical generation and supports iron-dependent lipid oxidation, thereby lowering the threshold for ferroptotic injury [16,29,64].

Iron and calcium homeostasis may also converge at the peri-implant bone interface, although their relationship should not be interpreted as a simple reciprocal concentration gradient. Calcium concentrations in the bone microenvironment are dynamically modified by mineral dissolution during osteoclastic resorption, transmembrane Ca^2+^ transport, and intracellular release from endoplasmic-reticulum and mitochondrial stores. RANKL-dependent osteoclastogenesis itself requires Ca^2+^ signaling, including IP_3_-dependent Ca^2+^ release, calcineurin activation, and NFATc1 signaling, whereas iron uptake primarily supports mitochondrial bioenergetics and the cytoskeletal machinery required for efficient resorption [66,67]. At the cellular level, the two ion systems may converge through redox and mitochondrial mechanisms: iron-dependent ROS can disturb Ca^2+^ handling, while excessive Ca^2+^ influx or mitochondrial Ca^2+^ loading can amplify ROS generation, membrane lipid oxidation, and ferroptotic susceptibility in experimental models [68].

Accordingly, local Fe and Ca gradients may coexist and dynamically influence bone-cell behavior, but direct spatial coupling between Fe and Ca concentration gradients has not yet been demonstrated in human dental peri-implant tissues. Their distributions are likely governed by different processes: iron by hemorrhage, erythrocyte turnover, macrophage sequestration, transport, and clearance, and calcium by mineral dissolution, cellular transport, and bone-remodeling activity. Simultaneous spatial mapping of labile iron and calcium, ideally combined with ferroptosis markers and cell-type identification, would therefore be required to determine whether these ionic gradients interact directly at the peri-implant interface.

### 4.4. Ferroptosis of Bone Cells and the Link to the RANKL/OPG Axis

The mechanistically decisive point is that ferroptotic dysfunction of bone cells can alter remodeling rather than merely reducing cell number. In osteoblasts, iron overload and loss of GPX4/FTH1 activity suppress differentiation and mineralization, and these effects can be attenuated by deferoxamine or ferrostatin-class inhibitors in skeletal models [18,69]. Importantly, direct particle-related evidence is now available: titanium particles induced GPX4 transcriptional repression, osteoblast ferroptosis, and peri-implant osteolysis in experimental models, and pharmacological or epigenetic rescue of GPX4 reduced the osteolytic phenotype [19]. This establishes a titanium-particle–ferroptosis connection in experimental implant osteolysis, although it does not yet demonstrate the same mechanism in human dental peri-implant bone. In osteocytes, broader skeletal studies indicate that ferroptotic stress and disturbed Nrf2 signaling can increase RANKL-related osteoclastogenic output and shift the RANKL/OPG balance toward resorption [70,71].

The available evidence points to a mechanistic sequence that can be reconstructed from separate experimental observations. Titanium particles suppress GPX4 and induce ferroptotic injury in osteoblasts, which impairs osteogenic differentiation and mineralization [18,19,69]. In skeletal models, ferroptotic stress in osteocytes alters their regulatory function: RANKL output rises and the RANKL/OPG balance shifts toward osteoclastogenic signaling [70,71]. Greater RANKL availability, in turn, drives osteoclast differentiation and activation, linking ferroptotic dysfunction of bone-forming and bone-regulating cells to increased resorption. Each component of this pathway has been documented individually, yet none of the studies has shown it to operate as a continuous causal sequence in human dental peri-implant tissue. Validating this pathway directly will require evidence at several levels. Within identified osteoblasts or osteocytes, human peri-implant specimens would need to show titanium degradation products, increased labile iron or iron accumulation, oxidized phospholipids, and impaired GPX4/SLC7A11 defenses co-localizing in space. Attributing this to ferroptosis further depends on compatible ultrastructural features and on excluding apoptosis, pyroptosis, and necroptosis through complementary molecular markers [48,49,50]. Beyond demonstrating the injury itself, one would need to show that ferroptotic bone-cell damage coincides with a local rise in the RANKL/OPG ratio and enhanced osteoclast activity in the same microenvironment. Causal confirmation ultimately rests on experimental or ex vivo models in which blocking ferroptosis—via ferrostatin-class inhibitors, iron chelation, or targeted restoration of GPX4 signaling—attenuates both the RANKL/OPG shift and the ensuing osteoclast-mediated bone loss. Absent these linked observations, the titanium–ferroptosis–RANKL/OPG pathway remains an experimentally supported hypothesis rather than an established mechanism of human dental peri-implant bone loss.

### 4.5. Convergence with Inflammasome and Macrophage Signaling

The lipid–ferroptosis axis intersects innate-immune signaling, but the direction and magnitude of this interaction are context dependent. Oxidized phospholipids may prime, activate, or in some settings suppress inflammasome-related responses depending on their molecular structure, concentration, carrier proteins, and cellular target [37,41]. Reactive aldehydes such as 4-HNE can modify signaling proteins and alter NF-κB activity, while ferroptotic cells may release HMGB1, oxidized lipids, and other damage-associated signals that recruit or activate macrophages [48,72,73,74]. It is therefore more accurate to describe ferroptosis as immunogenic or pro-inflammatory in selected settings rather than intrinsically inflammatory in every tissue. Similarly, macrophage responses should not be reduced to a rigid M1/M2 dichotomy; peri-implant macrophages occupy dynamic activation states shaped by particle burden, microbial products, lipid mediators, oxygen tension, and efferocytic capacity. Within the proposed framework, oxidized lipids constitute one set of upstream signals that can reinforce inflammasome activity and osteoclastogenic cytokine networks.

### 4.6. Osteoclastogenesis as the Endpoint

**Figure 2:**
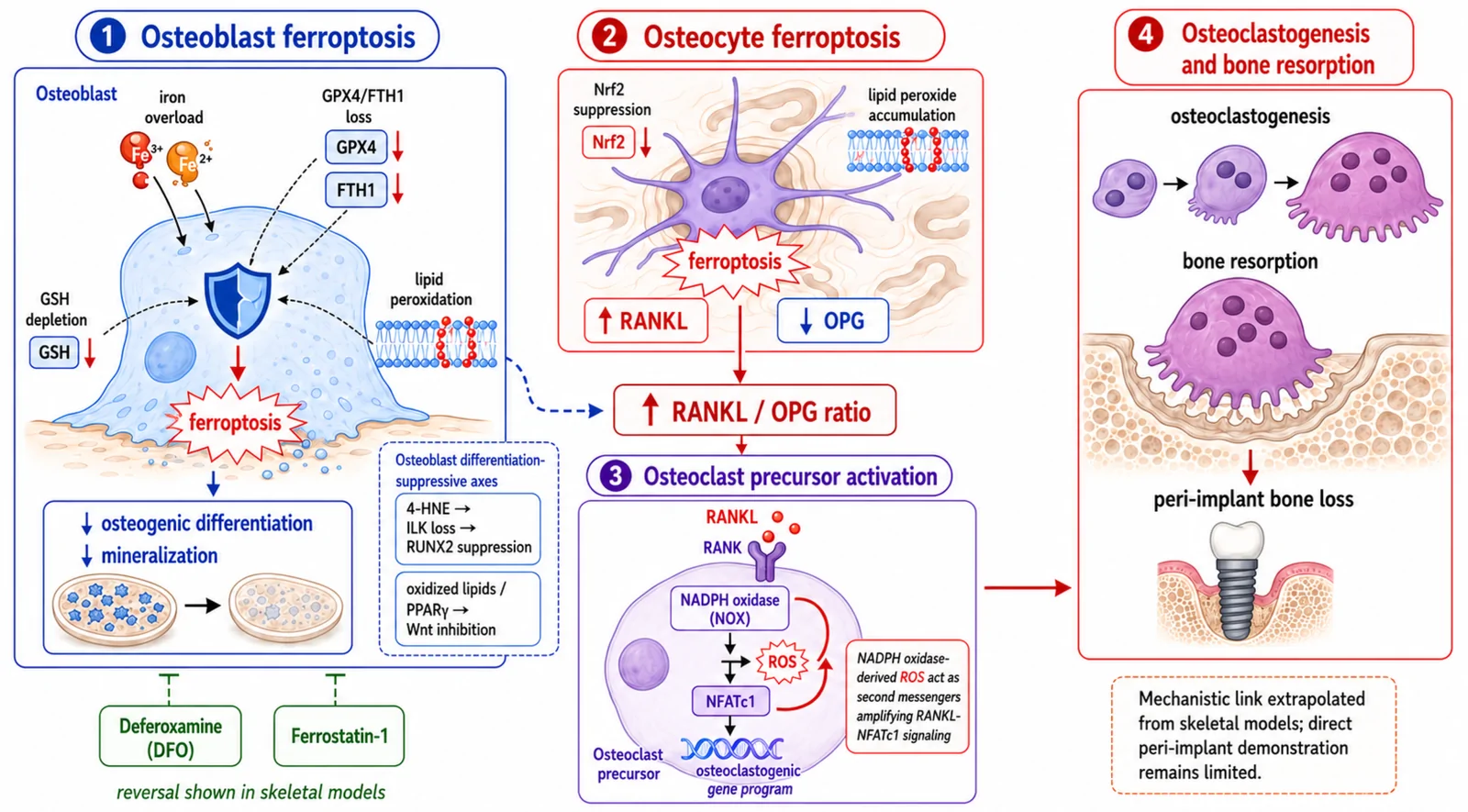
Cellular mechanisms linking ferroptotic stress to RANKL/OPG imbalance and peri-implant bone resorption. In osteoblasts, iron overload and impairment of GPX4/FTH1-dependent defenses promote lipid-peroxide accumulation, ferroptotic injury, and reduced mineralization. In osteocytes, ferroptotic and redox stress may increase RANKL-related osteoclastogenic signaling and alter the RANKL/OPG balance. NADPH-oxidase-derived ROS further amplify RANKL–NFATc1 signaling in osteoclast precursors, whereas the 4-HNE–ILK–RUNX2 and oxidized-lipid–PPARγ–Wnt pathways suppress osteoblast differentiation. Experimental rescue with deferoxamine or ferrostatin-class inhibitors supports the functional involvement of iron-dependent lipid peroxidation in skeletal models. The complete sequence has not yet been demonstrated in human dental peri-implant tissue. Red arrows indicate pro-ferroptotic or pro-osteoclastogenic signaling, including directional changes in pathway components, whereas black arrows indicate local molecular or cellular progression within individual mechanistic modules. Blue dashed arrows indicate proposed mechanistic links between ferroptotic bone-cell dysfunction and altered RANKL/OPG signaling. Green dashed blunt-ended lines indicate inhibitory or rescue effects associated with deferoxamine and ferrostatin-1. The colored panel borders and headings are used to distinguish individual mechanistic modules and do not indicate the strength of evidence. Original schematic developed by the authors based on published evidence [18,19,36,40,69,70,71,75,76,77,78]. Created with BioRender.com.

Under the present thesis, oxidative bone loss and osteoclastogenesis are recast not as parallel consequences of ROS but as the terminal readout of the lipid-ferroptosis axis. ROS act as second messengers in RANKL signaling, with NADPH-oxidase-derived superoxide promoting NFATc1 induction and osteoclast differentiation [75,76], while 4-HNE- and PPARγ-mediated suppression of RUNX2 and Wnt signaling simultaneously restrains bone formation [36,40]. These are the same endpoints reached when osteocyte and osteoblast ferroptosis shift the RANKL/OPG ratio toward resorption [70,71]. Presenting osteoclastogenesis as the endpoint of a defined lipid-ferroptotic pathway—rather than as a generic ROS effect—is a core distinction of this review. The cellular convergence of these events on osteocyte-driven RANKL output is depicted in Figure 2 (Section 9), whereas the principal molecular nodes and their evidentiary basis are consolidated in Table 3.

## 5. Microbial Contributions to the Lipid–Ferroptosis Substrate

Peri-implant dysbiosis and titanium release are reciprocally linked: microbial acidification, extracellular metabolites, and biofilm-associated electrochemical gradients can destabilize the passive TiO_2_ layer, whereas released titanium species may alter microbial community structure and host–microbe interactions [6,79,80,81]. Within the present framework, the microbial contribution is not simply a parallel etiology. Lipopolysaccharide and other microbial patterns prime NF-κB and inflammasome signaling; neutrophil and macrophage respiratory bursts increase extracellular oxidant production; acidic pH can alter metal speciation and corrosion kinetics; and bleeding or tissue ulceration provides heme-derived iron. These processes increase the supply of radical initiators and catalytic substrates needed for membrane lipid oxidation. At the same time, oxidized phospholipids and ferroptotic-cell products may impair barrier repair and perpetuate leukocyte recruitment, establishing a feed-forward loop between dysbiosis, material degradation, and redox injury. The available evidence supports mechanistic convergence, but direct demonstration that microbial dysbiosis promotes ferroptosis in peri-implant osteoblasts or osteocytes remains absent.

## 6. Systemic and Local Correlates of the Lipid–Ferroptosis Axis

### 6.1. Circulating and Local Biomarkers of Lipid and Nitrosative Oxidation

If oxidized-lipid signaling operates at the dental peri-implant interface, its biochemical footprint should be detectable in local fluids and tissues. Studies and evidence syntheses have reported altered oxidative-stress indices in peri-implant disease, although matrix type, sampling procedures, smoking, diabetes, periodontal status, and disease definitions introduce substantial heterogeneity [38,39]. A recent cross-sectional study measuring HIF-1α, GPX4, and MDA in peri-implant crevicular fluid found increased GPX4 and lower MDA in diseased groups relative to peri-implant health, suggesting a possible hypoxia-associated protective response rather than unopposed ferroptotic activation [82]. This finding is important because it challenges a simple linear model in which peri-implant inflammation necessarily produces GPX4 failure and increasing MDA. MDA, 4-HNE, F2-isoprostanes, protein carbonyls, and total antioxidant capacity should therefore be treated as contextual redox readouts, not stand-alone ferroptosis biomarkers. A more specific signature would require concordant evidence of oxidized PUFA-phospholipids, altered GPX4/SLC7A11/GSH function, increased labile iron, compatible ultrastructure, and pathway-selective rescue. Human evidence currently supports redox and ferroptosis-related signaling as an emerging area of investigation, not confirmed ferroptotic death in peri-implant bone cells.

### 6.2. The Dose Problem and Its Interpretation

A recurring limitation in extrapolating from experimental data is that many in vitro studies employ particle concentrations exceeding those plausibly encountered clinically, which risks overstating cytotoxic and ferroptotic potential [4,83]. Systemic titanium concentrations measured in implant patients are generally low, and titanium is highly insoluble and only slowly mobilized [84]. The reasonable interpretation is therefore not that titanium routinely drives systemic ferroptotic injury, but that the local peri-implant microenvironment—where particle burden, iron availability, and chronic inflammation concentrate—represents the plausible site for a lipid-ferroptotic effect. Throughout this review, mechanistic claims are intended to apply to this local context and not to systemic exposure.

### 6.3. Hypersensitivity Versus Redox-Driven Intolerance

Adverse titanium reactions are frequently investigated through the lens of metal allergy, using patch testing or lymphocyte transformation tests, which assess antigen-specific adaptive immunity and correlate poorly with implant outcomes [85,86]. We have previously argued that many such reactions are better understood as redox-driven innate immune intolerance rather than classical hypersensitivity [1]. That argument is not repeated here; it is relevant to the present thesis only in that a lipid-ferroptotic effector mechanism provides a concrete biochemical substrate for the “intolerance” phenotype that allergy testing cannot detect.

### 6.4. Orthopedic Wear-Debris Osteolysis as a Mechanistic Model

The most direct experimental analogue for titanium-associated bone loss comes from orthopedic wear-debris osteolysis, where particle burden is higher and mechanisms have been studied for decades. This literature established that metal and polymer particles drive macrophage activation, inflammasome signaling, and RANKL-mediated osteoclastogenesis culminating in periprosthetic bone loss [87,88]. Iron biology is increasingly implicated: developing osteoclasts upregulate TFR1 and depend on iron uptake, linking local iron availability to resorptive capacity [65]. The orthopedic model is therefore valuable both as precedent for particle-driven osteolysis and as the context in which iron- and lipid-dependent bone-cell death has begun to be characterized—supporting, by analogy, the plausibility of a comparable axis at the dental peri-implant interface while underscoring that the two settings differ in particle load and mechanical environment.

### 6.5. Host Susceptibility: Iron Status and Antioxidant Reserve

A distinctive and testable implication of the framework is that host factors governing iron handling and lipid-peroxide defense should modulate susceptibility. Conditions associated with systemic or local iron overload, chronic inflammation, or diminished GPX4/Nrf2 capacity would be predicted to lower the threshold for ferroptotic bone-cell death [65,70]. This reframes patient risk stratification in terms not previously emphasized for titanium implants: beyond smoking and diabetes, iron status and antioxidant reserve become candidate modifiers. These predictions are hypotheses generated by the framework, not established clinical risk factors, and are offered to direct future investigation.

### 6.6. A Balanced Interpretation

The evidence assembled here supports a graded interpretation. Titanium-particle–induced osteoblast ferroptosis is supported in experimental osteolysis models [19], ferroptosis is established as an iron- and lipid-dependent process in skeletal cells, and human peri-implant fluids show changes in GPX4 and lipid-peroxidation markers [82]. What remains unproven is whether ferroptosis occurs in human dental peri-implant osteoblasts or osteocytes and how much it contributes relative to apoptosis, pyroptosis, necroptosis, hypoxic adaptation, and conventional inflammatory osteoclastogenesis. The framework should therefore be read as an evidence-informed mechanistic model with directly supported experimental components and a still-hypothetical extension to the human dental peri-implant setting.

## 7. Methodological Limitations and the Evidentiary Gap

### 7.1. Absence of Direct Peri-Implant Ferroptosis Data

The central limitation is no longer a complete absence of titanium-related ferroptosis evidence, but the lack of direct demonstration in human dental peri-implant bone or soft tissue. Titanium-particle models of experimental peri-implant or periprosthetic osteolysis have shown GPX4 repression and osteoblast ferroptosis [19], and human peri-implant crevicular-fluid studies have begun to measure GPX4 and lipid-peroxidation markers [82]. However, fluid biomarkers cannot identify the dying cell type, establish iron dependence, or prove that ferroptosis contributes causally to clinical bone loss. Histological and spatial studies of human peri-implant specimens—combining oxidized-phospholipid mapping, GPX4/SLC7A11 status, iron localization, cell-type markers, and ultrastructural assessment—are therefore required.

### 7.2. Limitations of Experimental Models

Much mechanistic evidence derives from in vitro systems using particle concentrations, crystalline phases, and exposure kinetics that may not reflect the clinical setting [4,5]. Commercially available nanoparticles differ from clinically generated tribocorrosion debris in size distribution, aggregation state, crystalline phase (anatase versus rutile), oxidation profile, surface chemistry, and protein-corona formation, and each of these variables independently modifies cellular uptake and the downstream oxidative response, complicating extrapolation. A further confound is endotoxin: trace lipopolysaccharide adsorbed to commercial particles can by itself prime NF-κB and inflammasome signaling, so effects attributed to the metal may in part reflect microbial contamination unless endotoxin-free preparations are used [83].

Two additional mismatches deserve emphasis. First, tribocorrosion in vivo releases not only particles but a continuous flux of dissolved ions, and the ionic and particulate fractions can exert distinct—sometimes opposing—biological effects that single-material in vitro exposures rarely reproduce. Second, animal models of wear-debris osteolysis, most of them adapted from orthopedics, inform mechanism but differ from the dental peri-implant environment in mechanical loading regime, mucosal rather than periosteal soft-tissue coverage, anatomy, and the constant presence of an oral biofilm. These differences do not invalidate the models, but they mean that concentration thresholds, kinetics, and even the dominant cell-death mode observed experimentally must be re-established for the dental interface rather than assumed.

A further limitation is that most current systems are static and therefore cannot reproduce the dynamic mechanical and fluid conditions required to study titanium-associated redox injury and ferroptosis. In vivo, the dental implant–bone interface is continuously exposed to cyclic occlusal forces, mastication-induced micromotion, interstitial fluid movement, and temporally variable mechanical strain. These forces are not uniformly detrimental. Physiological mechanical loading is an essential regulator of peri-implant bone remodeling: mechanosensitive osteocytes translate strain into signals that promote osteogenic activity and coordinate osteoblast–osteoclast coupling. Adequate functional loading can therefore support bone formation and maintenance of osseointegration, whereas unloading favors disuse-related resorption. By contrast, excessive or poorly distributed loading, particularly when accompanied by micromotion, can produce local microdamage, osteocyte injury, altered RANKL/OPG signaling, and impaired osseointegration. Importantly, the evidence linking occlusal overload alone to peri-implant bone loss remains inconsistent, and its biological effects appear to depend strongly on the magnitude and distribution of strain and on the presence of concomitant inflammation [89,90].

Mechanical loading is also relevant to the material side of the proposed pathway. Repetitive contact, micromotion, and cyclic deformation can disrupt the protective titanium oxide layer and promote tribocorrosion, particularly in the chemically and microbiologically complex oral environment. Thus, mechanical forces may simultaneously provide a physiological osteogenic stimulus to bone and increase the release of titanium particles and ions when loading becomes associated with wear or interface instability. Static exposure models cannot reproduce this dual effect, because they separate the biological consequences of mechanotransduction from the mechanically driven generation of the degradation products that initiate redox stress [91,92].

Bleeding introduces a second dynamic component that is also largely absent from current experimental models. Peri-implant bleeding should not be regarded solely as a source of iron. Extravasated erythrocytes and subsequent erythrophagocytosis can locally increase heme-derived iron availability, but blood flow, interstitial fluid movement, extracellular scavenging systems, and cellular iron uptake can simultaneously redistribute or remove hemoglobin, heme, and soluble iron species from a given microregion. Consequently, the local labile-iron concentration and its spatial gradient are likely to fluctuate over time rather than remain constant. Macrophage sequestration and recycling of erythrocyte-derived iron further modify these gradients through ferritin storage and ferroportin-mediated export [63]. Direct measurements of time-resolved iron gradients in human peri-implant tissues are currently unavailable; therefore, the magnitude and duration of these effects remain to be established.

These considerations indicate that future peri-implant models should incorporate controlled cyclic loading together with dynamic fluid exchange and, where relevant, blood- or heme-derived iron sources. Such systems would better reproduce the simultaneous mechanobiological, tribocorrosive, and iron-transport processes that determine whether the peri-implant environment remains compatible with bone formation or shifts toward redox injury and ferroptotic susceptibility.

### 7.3. Causality and Confounding

Oxidized-lipid biomarkers such as MDA are non-specific and cannot by themselves confirm ferroptosis. A defensible assignment of ferroptosis in peri-implant models should combine: (i) increased lipid-peroxide burden measured with orthogonal methods, preferably including targeted oxidized-phospholipid analysis; (ii) evidence of iron dependence; (iii) impairment of GPX4- or system Xc^−^-centered defenses; (iv) compatible cellular or mitochondrial morphology; and (v) functional rescue by at least one chemically distinct ferroptosis inhibitor or iron chelator. Caspase activation, membrane rupture, or inflammatory cytokine release should be assessed in parallel to exclude dominant apoptosis, pyroptosis, or necroptosis [18,47,48,49,50]. Genetic manipulation of GPX4, ACSL4, SLC7A11, FSP1, or relevant iron-handling proteins would provide stronger causal evidence than marker expression alone. No peri-implant study has yet applied this complete evidentiary framework.

### 7.4. Underrepresentation of Lipidomic and Systems-Level Approaches

Peri-implant research has emphasized cytokine and histological endpoints, with comparatively little attention to lipidomics, oxylipin profiling, ferroptosis pathways, iron biology, or systems-level integration. This underrepresentation is itself a finding: the effector chemistry most proximate to bone-cell fate is among the least studied at the peri-implant interface. Cytokine panels and histomorphometry establish that inflammation and bone loss are occurring, but not the molecular species through which a redox signal is converted into impaired mineralization or excess resorption—precisely the step this review argues is decisive.

The gap is partly technical and partly conceptual. Technically, redox lipidomics capable of resolving individual oxidized phosphatidylethanolamine species requires mass-spectrometry workflows and sample handling that differ from the immunoassays routine used in periodontal research, and the small volumes recovered from peri-implant crevicular fluid impose additional analytical demands. Conceptually, ferroptosis and the pro-resolving lipidome entered mainstream biology only recently, so the field has not yet reframed its questions around them. Closing this gap is a prerequisite for testing the present framework at all, because the features that distinguish a ferroptotic program from generic oxidative injury are invisible to the endpoints currently in widest use.

### 7.5. Toward a Systems Framework

Addressing these gaps will require integrating redox lipidomics, iron mapping, ferroptosis-specific readouts, and osteoimmune profiling within the same peri-implant specimens and models, rather than studying each in isolation. The rationale for co-registration is that the axis proposed here is defined by relationships between variables—oxidized-phospholipid burden, labile iron, GPX4/SLC7A11 status, cell-type identity, and RANKL/OPG output—that lose their meaning when measured in separate cohorts. A high MDA value is interpretable only alongside the iron pool and antioxidant capacity of the same tissue; a fall in GPX4 signifies collapse or compensation depending on the accompanying hypoxic and lipid-peroxide context, as the divergent human findings in Section 6.1 illustrate.

Spatially resolved methods make this integration feasible. Combining oxidized-lipid or iron mapping with cell-type markers on the same section can localize the dying cell and its redox environment simultaneously, while single-cell and spatial transcriptomic approaches can situate ferroptosis-related programs within the broader osteoimmune landscape. Analyzed together within a single specimen, these readouts would allow the proposed lipid–ferroptosis axis to be confirmed, refuted, or refined—and, critically, to be distinguished from apoptosis, pyroptosis, and hypoxic adaptation rather than merely correlated with bone loss.

## 8. Future Directions

### 8.1. Redox Lipidomics and Pro-Resolving Mediators

Systematic lipidomic profiling of peri-implant biofluids and tissues—quantifying both pro-inflammatory oxidized lipids and SPMs—would establish whether peri-implant environments are biased toward injury or resolution, and whether that balance predicts clinical outcome [13,20]. The rationale is that the pro-inflammatory and pro-resolving branches of oxidized-lipid metabolism are generated by overlapping enzymatic machinery, so a single quantity such as total MDA cannot capture the direction of the response; what matters is the ratio of species that propagate injury (reactive aldehydes, hydroperoxy-phospholipids) to those that terminate it (lipoxins, resolvins, protectins, maresins). Liquid chromatography–tandem mass spectrometry now permits these families to be measured simultaneously in small-volume specimens such as peri-implant crevicular fluid, and comparable oxylipin panels have already been applied to gingival crevicular fluid in periodontitis, where a relative deficiency of SPMs tracks with non-resolving inflammation [43,44,45,46].

This resolution-oriented reading is not merely descriptive. In pre-clinical craniofacial and alveolar models, SPMs including resolvin E1, resolvin D2, lipoxin A4, and maresin 1 reduce inflammatory bone resorption and enhance regeneration, with a scoping review and meta-analysis reporting a significant increase in newly formed bone when SPMs are delivered to bone defects [93]. Maresin 1 additionally suppresses high-glucose-induced osteoblast ferroptosis through the NRF2/GPX4/SLC7A11 pathway [94], directly connecting the resolving branch to the anti-ferroptotic defenses developed in Section 4. A peri-implant lipidomic program should therefore report the injury-to-resolution balance as a candidate biomarker and, in parallel, test whether restoring that balance protects bone at the interface.

### 8.2. Testing Ferroptosis Directly

The proposed framework requires experimental systems capable of distinguishing ferroptosis from generalized oxidative injury while reproducing the dynamic conditions of the peri-implant interface. Initial studies should expose primary human osteoblasts, osteocyte-like cells, macrophages, and multicellular co-cultures to clinically generated tribocorrosion debris rather than exclusively to commercial TiO_2_ nanoparticles. Particle size distribution, surface chemistry, endotoxin contamination, protein-corona formation, and delivered cellular dose should be characterized. Ferroptosis should then be assessed using complementary endpoints, including C11-BODIPY or equivalent lipid-peroxidation probes, targeted oxidized-phospholipid lipidomics, labile Fe^2+^ imaging, GPX4 activity, GSH/GSSG, SLC7A11, ACSL4, FSP1, and transmission electron microscopy. Mechanistic specificity requires functional rescue with ferrostatin-1 or liproxstatin-1 and an iron chelator, ideally complemented by genetic perturbation of GPX4, ACSL4, or SLC7A11 [48,49,50].

Mechanical loading should be incorporated as an independent experimental variable rather than treated solely as a source of tribocorrosion debris. Bone-cell responses depend on the magnitude, duration, frequency, and mode of loading, and physiological and excessive mechanical stimulation may have fundamentally different biological consequences. Importantly, direct experimental evidence already indicates that compressive mechanical stress can modify ferroptotic signaling in osteoblasts: mechanical compression increased lipid peroxidation and altered GPX4 and ACSL4 expression, while ferrostatin-1 attenuated the associated loss of osteogenic activity [95]. Future peri-implant models should therefore compare unloaded controls with calibrated physiological and overload conditions and should assess ferroptosis and osteogenic readouts simultaneously. Mechanical variables should be reported quantitatively and linked to changes in GPX4/SLC7A11 activity, lipid-peroxide burden, RANKL/OPG signaling, osteogenic differentiation, and cell viability. Dynamic three-dimensional or mechanically stimulated culture platforms are particularly relevant because conventional static dental-implant models incompletely reproduce the biomechanical environment encountered in vivo [91].

Bleeding should likewise be modeled as a dynamic exposure rather than represented by a fixed increase in extracellular iron concentration. The biologically relevant variable is not simply the volume of blood present, but the resulting time-dependent exposure to erythrocytes, hemoglobin, heme, and subsequently released or recycled iron. Experimental systems could therefore incorporate graded erythrocyte or heme exposure together with controlled perfusion or medium exchange to reproduce different levels and durations of peri-implant bleeding. This distinction is mechanistically important because erythrophagocytosis can increase the macrophage labile iron pool, promote lipid peroxidation, and induce ferroptotic cell death when iron-handling capacity is exceeded [63]. Conversely, dynamic fluid exchange may redistribute or remove extracellular hemoglobin-, heme-, and iron-containing species, preventing the persistent local concentrations produced by static exposure systems. Whenever possible, labile iron should therefore be measured over time rather than at a single endpoint.

Multicellular models are particularly important under these dynamic conditions because loading and blood-derived iron are unlikely to affect all peri-implant cell populations in the same manner. Macrophages may sequester and recycle erythrocyte-derived iron, osteoblasts and osteocytes may undergo ferroptotic stress or alter osteogenic and RANKL/OPG signaling, and osteoclast-lineage cells may respond to changes in both iron availability and osteoimmune signals. Accordingly, future co-culture systems should quantify cell-type-specific responses rather than report only whole-culture averages. Spatially resolved imaging, cell sorting, or single-cell approaches could distinguish ferroptotic signaling in individual populations and determine whether macrophage iron handling protects neighboring bone cells or, under excessive iron burden, amplifies lipid-peroxidative injury.

A staged experimental strategy would therefore progress from static single-cell validation to mechanically loaded multicellular cultures, followed by perfused three-dimensional systems incorporating controlled erythrocyte/heme exposure and clinically relevant titanium debris. In vivo models should subsequently combine defined implant loading, microbial challenge, peri-implant bleeding or vascular injury, and time-resolved tissue sampling. Human validation should ultimately pair peri-implant fluid lipidomics with tissue immunohistochemistry, spatial iron mapping, cell-type-resolved ferroptosis markers, and clinical measurements of bone loss. Such an approach would determine not only whether ferroptosis occurs at the dental peri-implant interface, but also how mechanical forces, blood-derived iron, and intercellular crosstalk modulate its onset and biological consequences.

### 8.3. Iron- and Lipid-Targeted Protective Strategies

If the axis is confirmed, candidate interventions differ from generic ROS scavenging. Broad antioxidants quench radicals indiscriminately and have repeatedly disappointed in translation; the logic of ferroptosis instead points to node-specific targets: preservation of GPX4 activity, restraint of the labile iron pool by chelation or local iron modulation, interception of the propagation step by ferrostatin-class lipophilic radical-trapping antioxidants, and augmentation of pro-resolving lipid signaling. Each acts at a defined point in the pathway developed in Section 4 rather than on bulk oxidant load, which is what distinguishes this therapeutic frame from the redox-surface strategies discussed in our earlier work [1].

The pre-clinical support, while extrapolated from skeletal disease, is mechanistically concordant. In osteoblast and osteoporosis models, deferoxamine and ferrostatin-1 reverse iron-driven ferroptosis and restore mineralization and bone mass [18,64,94]; Nrf2/HO-1 activation sustains GPX4 and system Xc^−^ and protects osteogenesis under oxidative and diabetic stress [77,78]; and maresin 1 suppresses osteoblast ferroptosis through NRF2/GPX4/SLC7A11 while promoting regeneration [94], uniting the iron-, GPX4-, and resolution-targeted strategies on a single pathway. The principal translational caution is delivery: systemic iron chelation and global GPX4 manipulation carry off-target risks, so a peri-implant application would likely favor local or surface-tethered delivery, and any candidate must be validated against clinically relevant debris rather than model nanoparticles. These candidate strategies and their current evidence level are summarized in Table 4.

### 8.4. Host-Stratified Implantology

Iron status and antioxidant reserve may eventually contribute to host stratification, but they should not yet be presented as validated clinical risk factors. The framework predicts that conditions lowering the ferroptotic threshold—systemic or local iron overload, chronic inflammation, or diminished GPX4/Nrf2 capacity—should mark patients in whom a given particle and oxidant burden is more likely to translate into bone loss [65,70]. This is a genuinely novel axis of risk relative to the determinants usually emphasized in implantology, and it is testable: candidate markers include serum ferritin and transferrin saturation, local labile iron, and functional readouts of the GPX4/system Xc^−^ defense.

Realizing this clinically requires prospective evidence rather than mechanistic plausibility. The predicted modifiers must be shown to associate with longitudinal peri-implant bone change while controlling for established determinants—periodontitis history, smoking, diabetes, biofilm control, implant position, and prosthetic factors—and ideally to add predictive value beyond them. Until such data exist, iron and antioxidant status are hypotheses to be measured in future cohorts, not criteria for patient selection; presenting them prematurely as risk factors would repeat the overreach this review is at pains to avoid.

### 8.5. Redox-Responsive Biomaterials

Surface strategies that limit particle and ion release and modulate the local redox environment have been reviewed by us and others [1]. Within the present framework these approaches acquire a more specific rationale and a more specific test. If the effector chemistry that damages bone is iron-catalyzed phospholipid peroxidation culminating in ferroptosis, then the readout that matters for a redox-responsive surface is not merely lower total ROS or fewer released particles, but a measurable reduction in oxidized-phospholipid burden, preservation of GPX4/system Xc^−^ function, and suppression of ferroptotic signaling in adjacent bone cells—endpoints that current biomaterial evaluations rarely include.

To make the conceptual novelty transparent and avoid overlap with the authors’ previous redox-centered review, the distinct questions, mechanisms, and translational implications of the two articles are compared in Table 5.

## 9. Discussion

This review reframes titanium-associated peri-implant bone loss by shifting attention from the upstream redox trigger to downstream oxidized-lipid effectors. The central proposition is not that ferroptosis has already been established in human dental peri-implant tissues, but that it represents a biologically credible mechanism linking particle-associated oxidative stress with impaired osteogenesis and increased resorptive signaling. This proposition is strengthened by experimental evidence that titanium particles can repress GPX4 and induce osteoblast ferroptosis during implant-related osteolysis [19]. It is simultaneously constrained by human peri-implant fluid data suggesting that hypoxia may increase GPX4 and reduce MDA in some disease states [82]. The resulting model is therefore conditional and tissue-context dependent rather than a simple linear pathway.

Three features distinguish this framework. First, it assigns biochemical identity to the effector step by focusing on oxidation of defined membrane phospholipids and generation of reactive aldehydes rather than invoking undifferentiated ROS damage. Second, it integrates direct titanium-particle osteolysis evidence with skeletal studies of osteoblast and osteocyte ferroptosis, thereby separating established experimental mechanisms from dental extrapolation. Third, it incorporates counter-regulation through GPX4, FSP1, DHODH, hypoxic adaptation, and specialized pro-resolving mediators. Figure 1 summarizes the proposed material-to-tissue pathway, whereas Figure 2 illustrates its convergence on bone-cell dysfunction and RANKL/OPG-dependent osteoclastogenic signaling. Table 1, Table 2 and Table 3 organize the oxidized-lipid species, death-mode distinctions, and molecular nodes, and Table 4 separates candidate interventions according to their current level of evidence.

**Figure 3:**
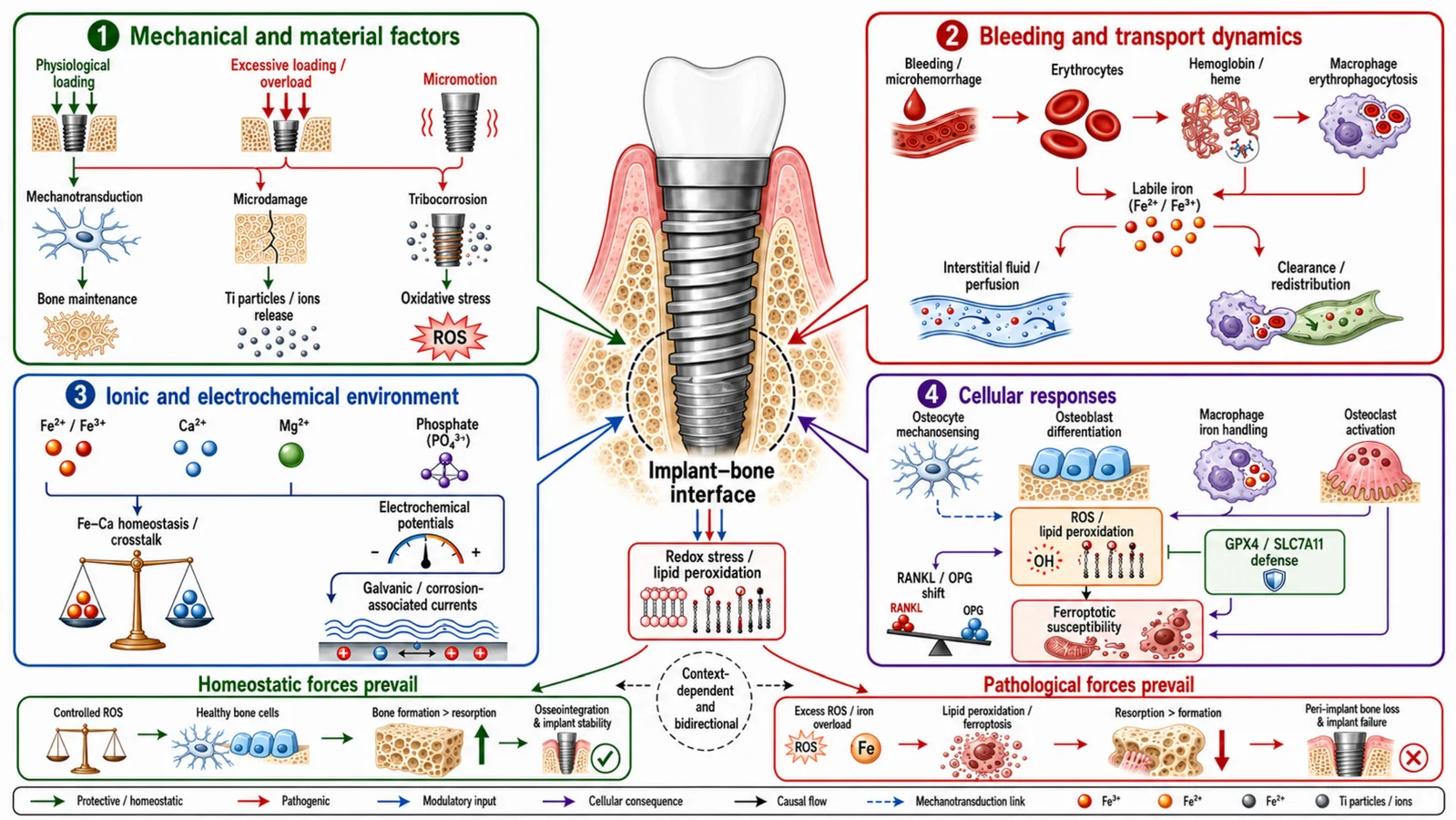
Dynamic physical, ionic, and electrochemical modulators of the peri-implant redox–ferroptosis axis. The dental implant interface is continuously influenced by cyclic mechanical loading, micromotion, fluid transport, bleeding, microbial activity, and electrochemical processes. Physiological loading promotes osteocyte mechanotransduction and supports bone maintenance, whereas excessive or poorly distributed loading and micromotion may increase microdamage and tribocorrosion, thereby enhancing titanium particle and ion release. Bleeding provides erythrocytes and heme-derived iron but, together with interstitial fluid transport and cellular iron recycling, may also redistribute or clear iron-containing species. Local Fe and Ca homeostasis can converge through redox, mitochondrial, and bone-remodeling pathways, although directly coupled Fe–Ca spatial gradients have not yet been demonstrated in human peri-implant tissue. Electrochemical potentials and corrosion-associated currents represent additional potential modulators of material degradation and cellular signaling. Green arrows indicate protective or homeostatic effects, red arrows indicate pathogenic processes, blue arrows indicate modulatory inputs, and purple arrows indicate cellular consequences. Black arrows indicate causal flow, whereas blue dashed arrows indicate mechanotransduction-related links. Dashed black connections denote context-dependent or potentially bidirectional relationships between the local interface environment and the balance between homeostatic and pathological outcomes. Original schematic developed by the authors based on published evidence [2,3,4,63,64,65,66,67,71,89,95,96,97,98]. Created with BioRender.com.

An important implication of this framework is that the lipid–ferroptosis axis should not be interpreted in isolation from the dynamic physical environment of the implant–bone interface. Mechanical loading, fluid movement, bleeding, local ionic composition, oxygen tension, microbial activity, and electrochemical phenomena can all modify the threshold at which a redox disturbance becomes biologically destructive (Figure 3). Their effects are also unlikely to be unidirectional. Physiological loading and controlled bioelectrical stimulation can support osteogenic signaling and bone maintenance, whereas excessive mechanical strain, persistent inflammatory stimulation, or abnormal electrochemical conditions may favor oxidative injury and resorption. The proposed pathway should therefore be viewed as a context-dependent network in which ferroptosis represents one possible downstream outcome when pro-oxidant inputs exceed the capacity of lipid-peroxide defense systems.

Electrical phenomena represent an additional, comparatively understudied component of the peri-implant microenvironment. Bone is electrically responsive tissue, and endogenous bioelectric potentials generated during deformation, ion transport, and cellular activity contribute to the regulation of osteoblast and osteocyte behavior. Experimental electrical stimulation has been reported to enhance osseointegration and bone regeneration, although the biological response depends strongly on current density, waveform, frequency, electrode configuration, and exposure duration [99,100]. Electrical signaling should therefore not be regarded as intrinsically detrimental; under controlled conditions, it can support osteogenic differentiation, angiogenic responses, and tissue repair.

A different situation may arise when electrical signals originate from electrochemical instability of the implant–prosthetic system. Titanium implants coexist with saliva and tissue fluids that function as electrolytes, and contact between metallic components with different electrochemical potentials can generate galvanic currents. Corrosion and tribocorrosion may likewise produce transient changes in surface potential and local current flow. These processes are coupled to disruption of the passive oxide layer and release of metal ions and particles, thereby linking electrical and chemical components of implant degradation [96,97]. Corrosion-associated electrical currents have been proposed as potential modifiers of osseointegration and peri-implant cell behavior, although their magnitude, spatial distribution, and biological significance in vivo remain poorly defined.

From the perspective of the present review, the most relevant unresolved question is whether such electrochemical signals can modify the lipid-peroxidation–ferroptosis threshold. Electrical and electrochemical perturbations can influence membrane polarization, ion transport, mitochondrial activity, and redox signaling, all of which may theoretically affect susceptibility to lipid-peroxide accumulation. However, direct evidence linking corrosion-generated electrical currents from dental implants to GPX4 failure, oxidized-phospholipid accumulation, or ferroptosis is currently lacking. Electrical signaling should therefore be considered a plausible upstream modulator of the proposed pathway rather than an established component of the titanium–ferroptosis axis.

The framework also clarifies why microbial and material-derived drivers act synergistically: both converge on iron-catalyzed lipid peroxidation as a common effector chemistry. It reconciles the clinical observation of measurable oxidative-stress biomarkers in peri-implant fluids with the difficulty of attributing pathology to any single upstream cause, by locating causation at a shared downstream node.

**Figure 4:**
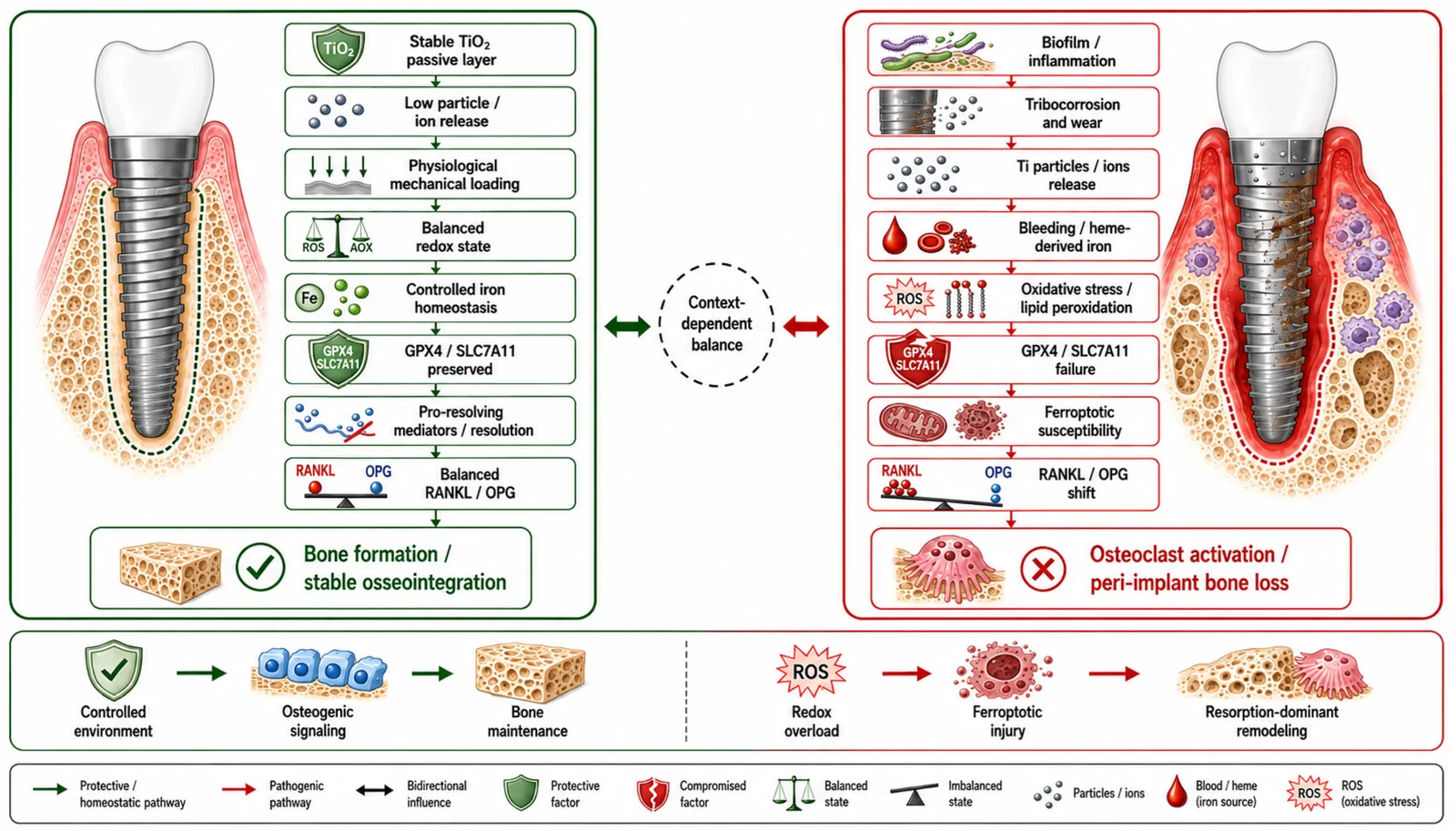
Conceptual comparison of a homeostatic osseointegrated interface and a pathological peri-implant microenvironment. Under stable conditions, preservation of the titanium passive layer, physiological mechanical loading, controlled redox and iron homeostasis, intact GPX4-dependent lipid-peroxide defenses, and pro-resolving signaling support balanced bone remodeling and osseointegration. In a pathological environment, microbial challenge, tribocorrosion, excessive or poorly distributed loading, recurrent bleeding, and titanium particle or ion release can converge on increased oxidative burden and lipid peroxidation. Failure of anti-ferroptotic defenses may then contribute to osteoblast or osteocyte dysfunction, altered RANKL/OPG signaling, osteoclast activation, and bone resorption. Ferroptosis is shown as a candidate downstream mechanism and has not yet been directly established in human dental peri-implant bone. Original schematic developed by the authors based on published evidence [1,2,3,4,5,6,18,19,39,63,70,82,89,95]. Created with BioRender.com.

The principal strength of this framework is therefore not the assertion of a single dominant pathway, but the integration of material degradation, redox chemistry, lipid biology, bone-cell signaling, and dynamic peri-implant factors into a testable mechanistic model. Titanium-particle-induced osteoblast ferroptosis provides direct experimental support for one segment of this model, whereas the involvement of osteocyte ferroptosis, local iron dynamics, mechanical loading, bleeding, and electrochemical signaling remains supported to different degrees by adjacent skeletal, biomaterial, and implant literature. These elements should not be assigned equal evidentiary weight. Rather, they define a hierarchy of hypotheses that can now be tested using spatially resolved human tissue analyses and dynamic experimental systems capable of integrating mechanical, electrochemical, and redox variables (Figure 4).

## 10. Conclusions

Titanium degradation products can impose a persistent oxidative burden on peri-implant tissues, but the downstream mechanisms that determine bone loss are heterogeneous and strongly context dependent. Experimental evidence demonstrates that titanium particles can induce GPX4 repression, osteoblast ferroptosis, and implant-associated osteolysis, supporting ferroptosis as more than a purely theoretical link between material degradation and impaired bone homeostasis. Nevertheless, direct confirmation of this pathway in human dental peri-implant bone is still lacking, and recent crevicular-fluid findings indicate that compensatory responses, including GPX4 upregulation under hypoxic conditions, may oppose ferroptotic lipid damage in some clinical settings.

Local iron availability should likewise not be considered in isolation. The peri-implant ionic environment contains Ca^2+^, Mg^2+^, phosphate, and other biologically active ions involved in mineralization, membrane signaling, cellular metabolism, and bone remodeling. Functional interactions between iron homeostasis and other ionic systems, particularly Ca^2+^-dependent bone-cell signaling, may therefore modify the biological consequences of changes in the labile iron pool [98]. However, direct spatial coupling between Fe and other ion concentration gradients has not yet been demonstrated in human peri-implant tissues. Simultaneous spatial mapping of iron together with calcium and other relevant ions would help determine whether such gradients contribute to ferroptotic susceptibility and altered bone remodeling.

Mechanical loading represents an equally important modifier of this environment. Physiological loading and effective force transfer are required for normal mechanotransduction, bone maintenance, and peri-implant bone formation, whereas excessive strain, micromotion, or poorly distributed loading may promote microdamage, tribocorrosion, and inflammatory bone remodeling [89]. Mechanical loading should therefore be viewed as a bidirectional regulator rather than simply as an additional damaging factor.

Oxidized-lipid signaling and ferroptosis should ultimately be considered components of a broader dynamic osteoimmune network shaped by material degradation, iron and ionic homeostasis, mechanical forces, inflammation, microbial activity, and local tissue transport. Future studies should integrate redox lipidomics, spatial iron and ion mapping, cell-type-resolved GPX4/SLC7A11 assessment, mechanical loading, ultrastructural analysis, and pathway-specific functional rescue. Such studies will determine whether the lipid-peroxidation–ferroptosis axis is a causal driver, a compensatory response, or a disease-stage-dependent modifier of peri-implant bone loss.

## Figures and Tables

**Table 1:** Oxidized-lipid mediators relevant to titanium-associated peri-implant bone pathology.

Lipid Species	Origin	Biological Action in Bone/Inflammation	Ref.
Malondialdehyde (MDA)	Terminal aldehyde of PUFA peroxidation	Protein/DNA adducts; established biomarker in peri-implant fluids	[13,39]
4-Hydroxynonenal (4-HNE)	Reactive α,β-unsaturated aldehyde; Michael adducts	Degrades ILK → disrupts RUNX2; NF-κB amplification; impairs osteoblasts	[14,40]
Oxidized phospholipids	Enzymatic/non-enzymatic PL oxidation	PRR/scavenger-receptor ligands; NLRP3 priming	[37,41]
Isoprostanes	Non-enzymatic arachidonate oxidation	Stable in vivo oxidative-stress markers; vasoactive	[42]
Specialized pro-resolving mediators	Enzymatic oxygenation (lipoxins, resolvins, protectins)	Pro-resolving; counterbalance injury (candidate protective branch)	[20]
Ferroptotic lipid peroxides	Iron-dependent PUFA-PL peroxidation	Execute ferroptosis; drive osteoblast/osteocyte death	[15,16]

PL, phospholipid; PRR, pattern-recognition receptor; ILK, integrin-linked kinase; PUFA, polyunsaturated fatty acid.

**Table 2:** Distinguishing ferroptosis from other regulated cell-death modes relevant to peri-implant tissue.

Mode	Core Mechanism	Morphology	Immune Character	Ref.
Ferroptosis	Iron-dependent lipid-peroxide accumulation; system Xc^−^–GPX4 collapse	Mitochondrial shrinkage, dense membranes, loss of cristae; intact nucleus	Context-dependent immunogenicity; may release DAMPs and oxidized lipids	[15,52]
Apoptosis	Caspase-3/8/9 cascade; intrinsic/extrinsic	Cell shrinkage, blebbing, apoptotic bodies, chromatin condensation	Usually immunologically silent when clearance is efficient; context dependent	[53]
Necroptosis	RIPK1/RIPK3/MLKL	Organelle swelling, membrane permeabilization	Pro-inflammatory	[53]
Pyroptosis	Inflammasome–caspase-1–gasdermin-D pores	Swelling, membrane rupture, IL-1β/IL-18 release	Strongly pro-inflammatory	[53,54]

DAMP, danger-associated molecular pattern. Only ferroptosis is iron- and lipid-peroxidation-dependent and specifically inhibited by ferrostatin-1/iron chelation, which provides the experimental basis for distinguishing it in peri-implant models (Section 7.3).

**Table 3:** Core nodes of the lipid–ferroptosis axis and their relevance to peri-implant bone loss.

Node/Molecule	Function in the Axis	Consequence for Bone	Ref.
PUFA/membrane lipids	Substrate for peroxidation	Source of MDA, 4-HNE, oxidized phospholipids	[13]
4-HNE	Reactive aldehyde; ILK binding, PPARγ ligand	Degrades ILK → disrupts RUNX2; suppresses Wnt → ↓ osteogenesis	[36,40]
Oxidized phospholipids	PRR/scavenger-receptor ligands; NLRP3 priming	Amplify sterile inflammation and inflammasome activation	[37,41]
Labile iron/TFR1	Fenton catalysis; osteoclast iron demand	Initiates peroxidation; permissive for osteoclastogenesis	[64,65]
System Xc^−^–GSH–GPX4	Central anti-ferroptotic defense	Collapse → lethal lipid-peroxide accumulation	[16,17]
Nrf2/HO-1	Sustains GPX4, SLC7A11	Failure precipitates osteoblast ferroptosis	[77,78]
Osteoblast ferroptosis	Iron-overload GPX4/FTH1 loss	↓ Mineralization; reversible by DFO/ferrostatin-1	[18,69]
Osteocyte ferroptosis	Nrf2 suppression → RANKL up	↑ RANKL/OPG → excess osteoclast activation	[70,71]

ILK, integrin-linked kinase; PRR, pattern-recognition receptor; DFO, deferoxamine; PUFA, polyunsaturated fatty acid. Most ferroptosis–bone evidence derives from skeletal and osteoporosis models; however, titanium-particle–induced GPX4 repression and osteoblast ferroptosis have been demonstrated in experimental implant osteolysis [19].

**Table 4:** Candidate lipid- and iron-targeted strategies for protecting peri-implant bone.

Strategy	Mechanism on the Axis	Evidence (Mostly Skeletal/Extrapolated)	Ref.
Iron chelation (DFO, deferiprone)	Lowers labile iron; blocks Fenton initiation	Reverses osteoblast ferroptosis and bone loss (skeletal models)	[18,64]
Ferrostatin-1/liproxstatin	Lipophilic radical trapping; halts lipid-peroxide propagation	Restores osteoblast viability and mineralization in vitro/in vivo	[18,77]
GPX4/system Xc^−^ support	Restores phospholipid-hydroperoxide clearance	Prevents ferroptotic osteoblast/osteocyte death	[17,77]
Nrf2/HO-1 activation	Transcriptional antioxidant induction sustaining GPX4	Protects osteogenesis under oxidative/diabetic stress	[78]
Pro-resolving mediators (SPMs)	Shift lipid balance toward resolution	Candidate; unproven in peri-implant setting	[20]
Redox-responsive surfaces	Reduce particle/ion release and local ROS	Readout should include lipid-peroxidation endpoints	[1]

DFO, deferoxamine; SPM, specialized pro-resolving mediator. All entries are extrapolated from skeletal or general models; none has been validated for peri-implant bone protection.

**Table 5:** Scope of this review relative to the authors’ prior work.

Dimension	Prior Work [1]	Present Review
Central question	Why the interface becomes redox-dysregulated (upstream trigger)	How the redox signal destroys bone (downstream effector)
Organizing thesis	Oxidative stress + antioxidant failure as unifying driver	Oxidized-lipid signaling and ferroptosis as effector arm
Alloy focus	Ti-6Al-4V; aluminium/vanadium redox activity	Titanium broadly; lipid/iron chemistry at the interface
Key mechanisms	ROS generation, NADPH oxidase, NLRP3, M1/M2, intolerance vs. allergy	Lipid peroxidation, 4-HNE–RUNX2, system Xc^−^–GPX4, iron, osteocyte ferroptosis–RANKL
Therapeutic frame	Redox-aware surfaces; antioxidant strategies	Iron chelation, ferrostatin, GPX4/Nrf2 support, pro-resolving mediators
Relationship	–	Builds on and explicitly cites the prior work; does not repeat it

## Data Availability

No new data were created or analyzed in this study. Data sharing is not applicable to this article.
